# Mechanical Metamaterials on the Way from Laboratory Scale to Industrial Applications: Challenges for Characterization and Scalability

**DOI:** 10.3390/ma13163605

**Published:** 2020-08-14

**Authors:** Sarah C. L. Fischer, Leonie Hillen, Chris Eberl

**Affiliations:** 1 Fraunhofer IZFP Institute for Nondestructive Testing, 66123 Saarbrücken, Germany; Leonie.hillen@izfp.fraunhofer.de; 2 Fraunhofer IWM Institute for Mechanics of Materials, 79108 Freiburg im Breisgau, Germany; chris.eberl@iwm.fraunhofer.de; 3 Department of Microsystems Engineering, University of Freiburg, 79110 Freiburg im Breisgau, Germany

**Keywords:** mechanical metamaterials, industry, scalability, characterization, processing-microstructure-property relationship

## Abstract

Mechanical metamaterials promise a paradigm shift in materials design, as the classical processing-microstructure-property relationship is no longer exhaustively describing the material properties. The present review article provides an application-centered view on the research field and aims to highlight challenges and pitfalls for the introduction of mechanical metamaterials into technical applications. The main difference compared to classical materials is the addition of the mesoscopic scale into the materials design space. Geometrically designed unit cells, small enough that the metamaterial acts like a mechanical continuum, enabling the integration of a variety of properties and functionalities. This presents new challenges for the design of functional components, their manufacturing and characterization. This article provides an overview of the design space for metamaterials, with focus on critical factors for scaling of manufacturing in order to fulfill industrial standards. The role of experimental and simulation tools for characterization and scaling of metamaterial concepts are summarized and herewith limitations highlighted. Finally, the authors discuss key aspects in order to enable metamaterials for industrial applications and how the design approach has to change to include reliability and resilience.

## 1. Introduction

Metamaterials are three-dimensionally architected materials whose properties are not only governed by their bulk properties, but also rather by their internal geometrical design [1]. In contrast to foams, metamaterials are composed of “unit cells”: three-dimensionally arranged, well defined geometrical building blocks [2,3,4]. Feature sizes range from the macroscopic elements in the millimeter range down to the nanoscale—depending on the manufacturing strategy [5,6,7,8,9,10]. The term metamaterials has been introduced by Rodger M. Walser in 1999 [11,12]. It is derived from the Greek word “meta” which means “beyond” to symbolize materials that have properties beyond those achievable with traditional materials [13]. The word has since been used with many different nuances as the research field has grown and gained attention [13,14,15].

**Figure 1:**
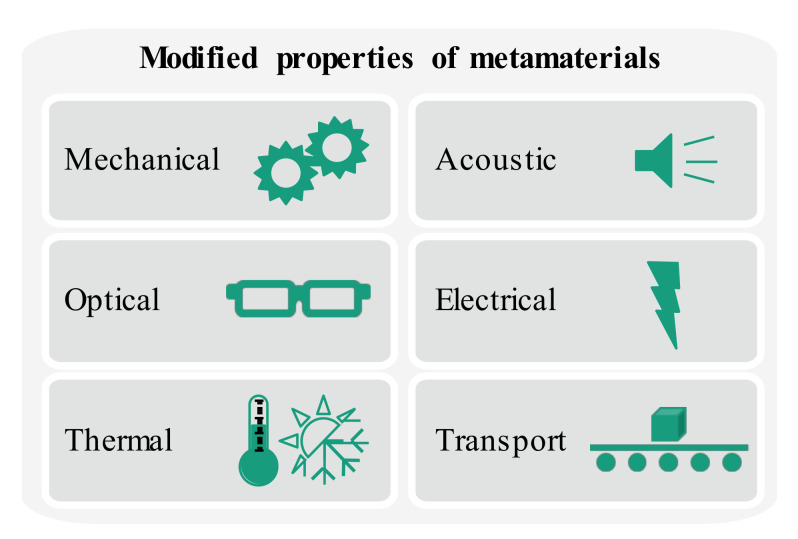
Overview of different metamaterial classes according to the modified properties of the material.

Metamaterials are typically subdivided according to the properties manipulated through their architecture. This includes for example mechanical [2,16,17], thermal [18,19,20,21,22], optical [23,24,25,26], electronical [11,27], acoustic [28,29,30] or transport [5,31] properties (Figure 1). Integrating the inner geometrical architecture of materials as a new dimension represents a significant expansion of the design space for materials and has enabled for example, cloaking mechanisms, shape-morphing or high strength-to-density ratios [1,5,19,32,33]. In many ways, these materials could be at the heart of innovative solutions for our society’s challenges including sustainability, mobility and energy storage.

In mechanical metamaterials the concerted reaction of the unit cell system behaves like similar to a mechanical continuum to attain the desired functionalities. Unit cells are composed of intricate, high aspect ratio elements to realize enable tailored deformations such as folding mechanisms, rotating elements or other functional properties [5]. The large deformation of the inner structures under external loads is at the heart of mechanical metamaterials and needs detailed understanding, both to realize attain the desired functionality and also to understand the impact of defects on the material’s lifetime.

In this paper, the authors discuss the upcoming challenges when expanding the classical processing-microstructure-property relationship through mechanical metamaterials. It offers an overview of the design space from an application’s perspective. Bridging the gap between research on metamaterials and their translation to technical applications will require a considerable effort. The authors did not include an exhaustive overview about all concepts and research work from the community and refer the reader to the available resources ([1,6,14,15,34,35,36,37] and many more). Instead, the authors present a novel angle of observation by addressing the challenges related to translating metamaterials from laboratory concepts to industrial concepts to benefit our society as a high-level overview. The aim is to stimulate discussion in the community and point out aspects of metamaterials that need attention from researchers in the future to enable scaling the technologies.

## 2. Current Design Space for Mechanical Metamaterials

**Figure 2:**
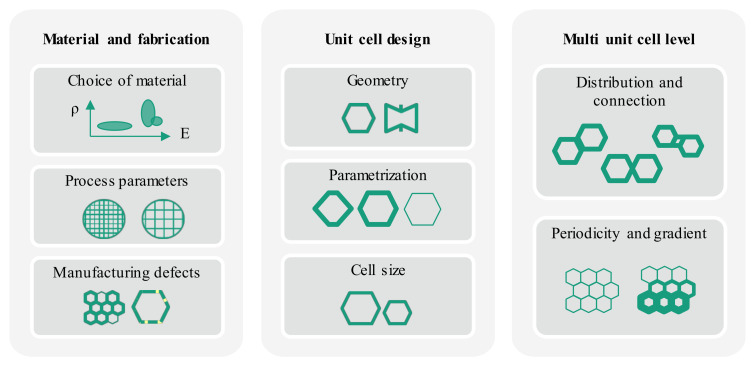
Design space of mechanical metamaterials: parameters originating from material and fabrication, unit cell design and multi unit cell level architecture.

While simplifications such as linear behavior at small strains are often-times valid for a first approximation of conventional materials, mechanical metamaterials require a higher level of understanding of their properties. Mechanical properties of metamaterials result from a superposition of many different factors: bulk material, manufacturing parameters, defects from fabrication, unit cell geometry, cell size, parametrization, unit cell distribution, connection and periodicity (Figure 2). These factors will be illustrated in more detail individually in the following subsections [1,3,38,39,40].

### 2.1. Choice of Materials, Manufacturing Process and Manufacturing Parameters

The vast majority of published metamaterials are made out of polymers as they can generally accommodate larger deformations [2,3,16,27,37,41,42,43]. However, it is important to remember that polymers can exhibit viscoelastic properties that will influence the overall mechanical properties, especially under loading conditions [44].

The most common materials used are polymeric photoresists (e.g., acrylic photoresist with an approximate Young’s Modulus of E=2.4 GPa), which can be processed using two-photon lithography [2,16,45]. This method enables fabrication of complex, from high resolution three-dimensional structures down to the micron-scale.

Other concepts rely on filament deposition using various thermoplastic filaments including shape memory polymers, polylactic acid (PLA) or acrylonitrile butadiene styrene (ABS) [5,46]. Another additive manufacturing concept relies on stereo lithography using elastomers [47,48].

Common to all additive manufacturing techniques, the resulting parts are highly dependent on process parameters and exhibit anisotropy or defects. Residual stresses, preferred orientations of polymer chains and local variations in the cross-linking density are common factors that have to be taken into account especially for scaling model systems [3,10,49].

Besides the well-known additive manufacturing techniques, a few less common techniques however, also show great potential. Hollow spheres made from a polymer reinforced with nanotubes are one example where Yuan et al. [50] achieved a metamaterial with exceptional energy absorption and tensile toughness due to the carbon nanotube networks and structural design. Furthermore, casting of silicone rubber is another approach found in literature used for 2.5 dimensional beam structures [41], while water jet cutting was applied to photo-elastic elastomer PSM-4 to generate regularly spaced holes within the specimen [51].

For applications the load bearing of polymers is often too low. Therefore, metals or ceramics need to be qualified for metamaterial. A few concepts for metallic mechanical metamaterials exist leveraging additive or subtractive manufacturing techniques (e.g., using computer numerical control (CNC) machining or a focused ion beam (FIB) [10]) to create patterned sheets of metal which can then be layered to form the overall structure [31,37,52]. Metal-polymer composite metamaterials are achieved by polyelectrolyte-brush-assisted electroless plating (ELP) of copper. Etching of the interior polymer structure leads to hollow metallic metamaterials [43].

Using coating technologies, even ceramic metamaterials have been explored [33,53,54]. Bauer et al. [45] also manufactured polymeric microarchitectures with two-photon lithography followed by a pyrolysis step to obtain carbon nanostructures, which were then coated with 10 nm thin alumina.

### 2.2. Unit Cells: Geometry and Parametrization

**Figure 3:**
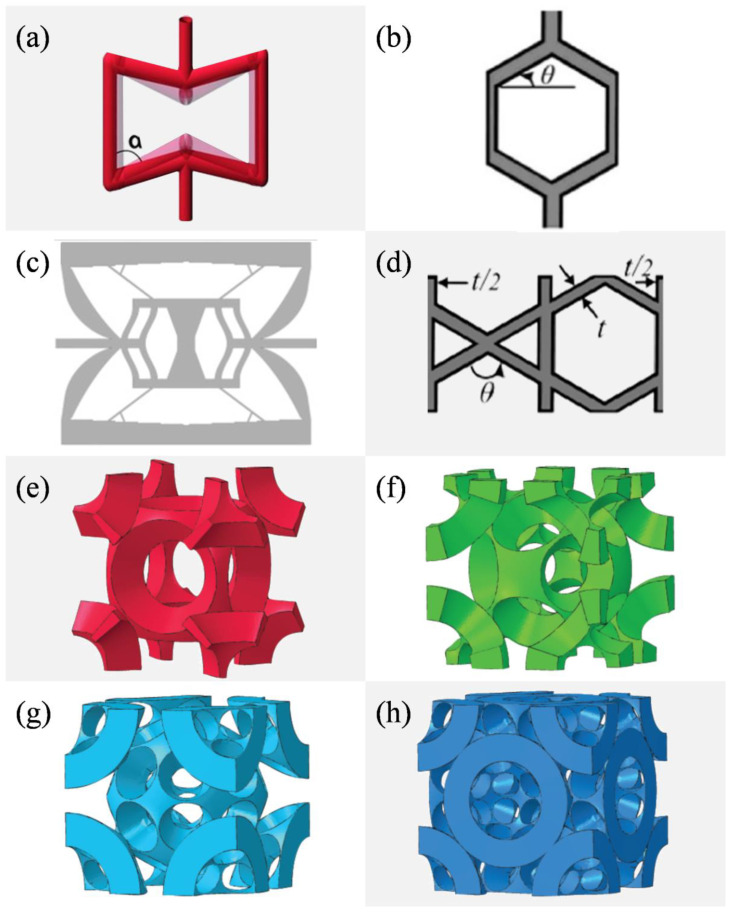
Overview of different unit cells which will be challenging to manufacture but propose very interesting properties for mechanical metamaterials: (**a**) basic bow tie [16], (**b**): honeycomb [3], (**c**): auxetic bow tie [2], (**d**): kagome based unit cell [3], (**e**–**h**): bucklicrystals with differing amounts of holes arranged body centered cubic (BCC) or face centered cubic (FCC) [57]: (**e**) 6-hole BCC, (**f**): 12-hole BCC, (**g**): 24-hole BCC, (**h**): 24-hole FCC. Figures are reproduced and adapted with permission from John Wiley and Sons [2,3,16,57].

Unit cells are the smallest repeating volume of intentionally designed geometrical features [14]. They often consist of thin, high aspect ratio trusses linked by joints [5]. Figure 3, while not claiming completeness, shows an overview of some mechanical metamaterial unit cells published in literature. There are many variations possible to alter properties at unit cell level. Depending on the intricacy of the design, they are determined by parameters such as the truss diameter, truss angle or height. The deformation mechanisms in the unit cells can largely be categorized as bending-dominated or stretching-dominated [3,5,53,55].

Through variation of unit cell parameters, the overall mechanical response of the system can be influenced. There are but also auxiliary factors to be considered such as density, preferential direction of deformation or failure mechanisms [4,41,50]. For more detailed information on the design of mechanical metamaterials, both regarding single unit cells and networks of unit cells, the reader is referred to the many review articles dedicated to this topic [1,5,6,35,37,56].

While many of the existing unit cell designs are intricate in their properties gained from their inner structure, the manufacturing of cubic meters of such materials have not been considered. The challenges arising here will necessitate a tight interaction between mechanics and processing experts in order to simplify the unit cell design; to make it fit for a specific manufacturing process. It is to be expected, that this effort will need also require optimization in both directions which only can be achieved by interdisciplinary research projects.

### 2.3. Unit Cell Scaling Effects: Size, Connections and Distribution

The deformation behavior of a single unit cell is not indicative of the global properties of a system [42]. The connections between unit cells are of utmost importance; they determine the transfer of forces between unit cells. The transfer of forces defines how external forces act on the material, for example localized forces, forces over a large area, forces from all sides, forces acting on certain preferred directions [39]. Therefore, the connections need to be designed with potential applications of the material in mind.

The number of unit cells can determine the overall deformation behavior. Depending on the architecture of the unit cell, an odd or even number of unit cells can change the way forces spread across neighboring cells and change the global behavior as shown by Coulais et al. [39] with a system of counter-rotating hinged squares. In addition, an important factor here is the number of cells per unit length in relation to the dimensions of a part. There is a critical number of unit cells necessary for them to emulate like a continuum and behave in a homogeneous way. [9,39,42]

Periodic arrangements of unit cells such as regular honeycomb or bow tie structured volumes account for the majority in metamaterial design, but there are also concepts with hierarchical, aperiodic or gradient-like distributions of unit cells [2,3,9,16,58,59]. Non-periodic arrangement is used to realize non-homogeneous deformation properties such as travelling waves or shape shifting aperiodic designs [60]. Gradient-like arrangements represent a continuous change in properties which can, for example, be realized by gradually changing certain features such as truss diameter [35,41] within the unit cell parameter space.

### 2.4. Manufacturing Defects

All manufacturing processes result in defects, flaws and discontinuities, including for example, porosity, anisotropy, in-plane and out-of-plane thickness-variation, voids or weakly adhered or misaligned joints. Defects, i.e., any deviation from the intended geometry, have a critical impact, altering material behavior and mechanical properties. The effect of defects varies with the different length scale of the unit cells. [1,3,49,50,53,61]

Failure or irreversible deformation of the structures can occur due to unintended defects in the material. However, they can also be a part of the functionality of the metamaterial and are purposely designed into the material: cuts (e.g., in kirigami structures [62,63,64]), folds and wrinkles (e.g., the miura fold patterns [65,66]) in sheets) or instabilities (e.g., to create bistability or even multi-state metamaterials [67,68,69] or snapping mechanisms [55]). Here, the defects serve as mechanisms, creating the unique feature of mechanical metamaterials.

In classical metallurgy, defects in materials are used to achieve certain properties, e.g., yield strength, toughness or an enhanced lifetime. This concept of using a defect to strengthen and make it more resilient can also be used at a mesoscopic scale as was shown by Pham et al. [70] by designing metamaterials similarly to a polycrystalline material. The volume consisted of differently oriented metamaterial crystals, which were connected by grain boundaries. The introduction of this hierarchy level expands the design space even further and can be used to toughen the typically brittle nature of single-material mechanical metamaterials. This is a rare example of the integration of aspects of resilience into the design of mechanical metamaterials that is crucial for translation to technical applications.

On the laboratory scale, many different manufacturing approaches, unit cell designs and unit cell arrangements are presented to produce macro- to nano-size systems [1,5,14,38,71,72,73,74]. They all have unique characteristics, limitations and process-induced flaws. However, only very few of them are directly translatable into larger scale manufacturing as fabrication speed and development time are usually not factors of high importance in the academic materials and mechanics world. In contrast, industry relies on fast turnaround times for economic reasons and also requires a much more thorough quality control of components with minimal production rejects. Translating metamaterial concepts from the laboratory bench to technical applications, several aspects need further attention:

- redesign of the system and its unit cells to enable large scale production
- development of design tools to simplify customization of metamaterials
- implementation of characterization methods to evaluate the quality of manufactured metamaterials
- validation of techniques and processes for predictive maintenance during the life-cycle
- design rules to improve resilience of metamaterials and models for life-time prediction.

Before resilience of metamaterials is discussed in more detail in Section 4, the two main strategies to explore the mechanical behavior of mechanical metamaterials, numerical simulation and experimental characterization, are summarized with respect to the above challenges.

## 3. The Role of Simulation in Understanding, Optimizing and Scaling Mechanical Metamaterials

Numerical simulations are a powerful tool for the design and optimization of mechanical metamaterials to manage the complexity of the parameter space. In this section, their present role will be described as well as desirable future evolution as metamaterials transfer from the laboratory bench to technical applications in industry.

### 3.1. Optimization of Metamaterials

All typical material modeling techniques and estimations of size effects are translated into metamaterials from classical materials in a first approximation [5]. Continuum mechanics methodology is based on the assumption that materials behave as a continuum; this can be a drastic simplification. Therefore data and model development need to be improved [75]. Analytical methods can provide additional insight for the fundamental design and description of metamaterials, where mechanical equivalent models can be established. However, the employment of numerical approaches is usually preferred, especially with regard to the scaling of the problem [2,16,38,58,75].

**Figure 4:**
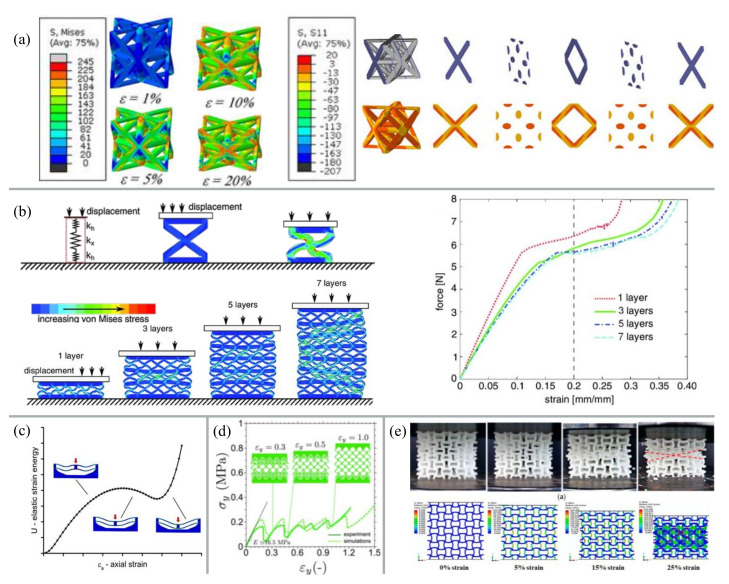
Examples of simulations as tool for topological and deformation optimization of metamaterial unit cells. (**a**) Simulation of Van Mises stresses in octet-truss unit cells under different strains (left) and stress distribution within the octet-truss at 1% strain (right) [78]; (**b**) Simulation of Van Mieses stress in unit cells showing deformation and collapse behavior [41]; (**c**) Simulation of elastic energy of a unit cell with bi-stable behavior under compression [2]; (**d**) Experiments and simulation of the stress-strain diagram of a sequentially snapping metamaterial [55]; (**e**) Experiment and simulation of a metamaterial under compression [79]. Figures are reproduced and adapted with permission from John Wiley and Sons [2,41,55,78] or are published under a Creative Common Open Access License [79].

Numerical simulations are applied to screen the parameter space on single unit cell level or are based on networks of unit cells (Figure 4). Variations of parametrization especially can be studied systematically to reduce the experimental work [72,76,77]. In addition to be used to study the influence of design parameters, simulations are also used to complement experimental work. For example, to show negative Poisson’s ratio or unconventional deformation behavior. There is merit in showcasing that the deformation behavior of intact structures without flaws can be predicted. But astonishingly little attention is given to any discrepancies observed between simulations and experiments [38,42,66]. Some publications vaguely attribute the differences to possible flaws and defects in the material, but it is not an integral part of the material development and is often overlooked [42]. Although the goal is the production of perfect materials without defects; samples in a research study can be selected this way. However, it is of fundamental importance to know about the effect of defects in materials, as they will inevitably occur when the processes are scaled up.

Not only defects innate in the manufacturing are of utmost importance, but a global understanding of the formation and propagation of defects in metamaterials during application, with respect to the influence on the overall functionality. Is there a critical number of unit cells that can fail in a network before the overall functionality is altered? How does the material behave with defects, does it still fulfill basic functionality or is there a risk for a certain application? Scientists could try to translate failure criteria from other research fields to further this understanding through numerical simulations. Another approach could be to incorporate defects in unit cells as one of the design parameters and to simulate networks with random distribution and controlled percentage of non-functional unit cells to assess their influence on overall behavior [59,79].

With regard to possible applications in industry, it is likely that the resilience of metamaterials is of equal importance as their actual functionality. After understanding the effect of defects in a numerical simulation-based approach, it is essential to develop the experimental tools to estimate the resilience of each part at beginning and during its lifetime. This will be addressed in more detail in Section 4 and Section 5.

### 3.2. Optimization of Metamaterials Systems

At a laboratory level, the structure can be simulated with greatest precision for research purposes in order to explore the parameter space. As soon as specific applications are targeted, the material is no longer isolated. It will become a part of a complex system of interacting parts and the highest priority of simulations is to predict system behavior appropriately. This requires a different approach to describe the metamaterial in a simplified, yet accurate way. Equivalent material models and homogenization can be used to increase computational efficiency.

### 3.3. Product Design for Applications

**Figure 5:**
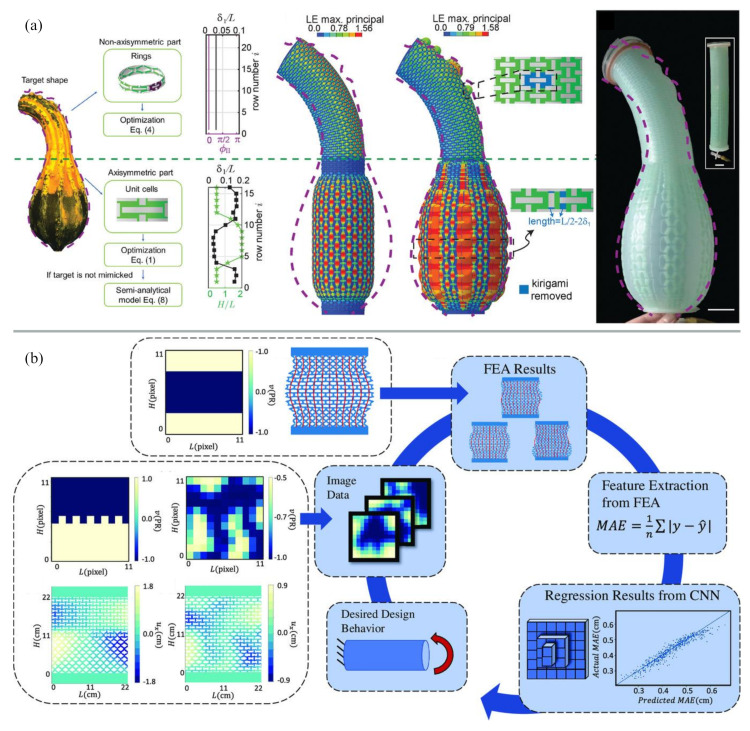
Examples of workflows for automated optimization of metamaterial systems. (**a**) Design of programmable inflatable actuators [80]; (**b**) optimization of auxetic material based on deep-learning [81]. Figures are reproduced and adapted with permission from John Wiley and Sons [80,81].

Looking at the materials from an application perspective, they offer too many design possibilities and it is impossible for the customer to create the perfect design, arrangement and manufacturing strategy for metamaterials without computational efforts. If metamaterials are to be the solution for industrial applications, having the dreamed of impact oft-quoted in publications, more important than an ever increasing number of novel unit cell geometries or higher resolution fabrication strategies, there is a huge need for a workflow for their design [80,81] (Figure 5). An important role of simulations here is to provide a toolbox that can benefit from the many existing concepts for unit cells and provides the user with a tool to preselect most suitable parameters for their application. The application needs to describe the practical limits for the optimization and, for example, contain the kind of mechanical behavior required, the amplitude of external forces, what materials can be used for and minimum requirements concerning strength and resilience.

Although there are a few research publications emerging on this topic [80,81] as shown in Figure 5, neighboring fields have addressed this much earlier. In computer science, metamaterials and additive manufacturing are considered accessible ways to modulate material properties to alter for example human perception or make “machines” [82,83,84]. This field has developed tools that enable a user to design and prototype their own metamaterials due to the focus on the products and usability [84,85].

## 4. The Role of Experimental Investigations in Understanding and Scaling Mechanical Metamaterials

Most currently applied methods to characterize mechanical metamaterials were developed for bulk materials (Table 1). Because the deformation behavior is so unique and important in mechanical metamaterials, multimodal experiments are usually conducted combining mechanical testing with an imaging technique.

### 4.1. Experimental Mechanics Methods

Uniaxial compression tests are the most common method of evaluating mechanical metamaterials. Uniaxial tensile tests are performed rarely, as current metamaterials are typically very brittle under tensile load. The experimental characterizations are performed with different pieces of equipment, depending on the size of the samples. This includes conventional universal testing machines (e.g., ADMET, Instron, etc.), nanoindentation (Hysitron, nanomechanics, etc.), in-situ stages in scanning electron microscopes (e.g., InSEM, Nanomechanics Inc.) or custom-built setups (Micromechanical Setup) [2,3,5,32,45,66]). Among these techniques, two approaches dominate the community. On the one hand, proof-of-concept studies on macroscopic samples on the scale of several millimeters unit-cell size are performed with uniaxial tensile testing. On the other hand, samples with dimensions in the micron- or sub-micron-scale fabricated by two-photon lithography are tested by higher-resolution devices [32,45,86]. Studies are mainly performed in the quasi-static range while the impact of dynamic or crash behavior is rarely considered.

All other mechanical tests are much less frequent and depend on the target properties of the metamaterials. Three-point-bending tests were performed on shape memory polymer honeycomb structures to evaluate the impact of different programming strains on the honeycomb structure [3].

Time dependent phenomena have been seldom considered for mechanical metamaterials [3]. Although the velocity of testing is varied in some uniaxial experiments, relaxation and creep behavior is rarely studied. Berwind et al. [2] applied and held several different loading states to their metamaterial on a micromechanical scale and observed that relaxation depends on base material, structure and further design parameters.

Cyclic experiments can also yield interesting information concerning repetitive mechanical loading of metamaterials and are used in a few research projects. [54]

There are some more exotic characterization methods arising from the interest in metamaterials in the field of human-computer interaction. As this community is very interested in human perception, experiments are created with human touch in mind, for example recreating a finger pushing a material locally, similarly to local indentation experiments. [87]

The main focus of mechanical experiments in publications is showcasing the deformation behavior of the fabricated unit cells or unit cell networks as a proof-of-concept, even though the latter are less thoroughly studied compared to the behavior of single unit cells. Very few publications target failure mechanisms and the impact of defects [70,88]. Random imperfections due to irregularities in fabrication have been studied in two-dimensional honeycomb-structures. Imperfections considered were missing and misaligned walls and non-uniform cell sizes [89,90,91]. Simone et al. [89] Chen et al. [91] and Li et al. [90] studied the impact of these defects on elastic modulus and compressive strength. Most of the research is still limited to validating the properties rather than fully characterizing the generated material. While validation of the metamaterials shows through documented proof that the material reproducibly fulfills the prior specified requirements, characterization goes one step further. Not only the functionality, but the overall properties of the material including reliability and life expectancy are considered.

### 4.2. Non-Destructive Characterization

Although one could argue that some of the experimental techniques presented above are non-destructive as long as the deformation is reversible, these are not methods that can easily be deployed to ensure the quality of a large volume of material. Thus, they are not considered as non-destructive in this paper. The characterization of metamaterials with truly non-destructive methods is currently mainly limited to imaging techniques [5].

Optical imaging techniques as well as scanning electron microscopy are used to accompany mechanical testing (see Section 4.1) [27,43,54,79,92]. X-ray tomography gives a three-dimensional insight into the geometry. This technique provides a way to quantify for example absolute dimensions, geometrical deviation from the designed model or defects. The data gained can also be used as an accurate 3D-model for simulations [57,93].

Not much attention has been dedicated to adapting non-destructive evaluation techniques beyond imaging capability, even though metamaterials have attracted interest in the non-destructive evaluation community as interesting novel sensor or actor materials. Indeed, they show promising properties concerning their interaction with ultrasound or electro-magnetic techniques [94,95,96,97]. Surprisingly, this knowledge has not systematically been transferred to characterizing the metamaterials.

Characterization based on non-destructive testing needs to progress to live up to the challenges of mechanical metamaterials. With the perspective of scaling manufacturing processes and application of the materials in industry, it is necessary to have non-invasive qualification and monitoring methods to gain quantitative information on mechanical properties and assess present defects to be able to guarantee resilience of the mechanical metamaterials in their targeted application.

Furthermore, to enable verification of the structural integrity of these complex materials, it is important to scale and adapt non-destructive testing to monitor manufacturing of mechanical metamaterials. The hierarchical and complex nature of mechanical materials can lead to interference with non-destructive probes or [98] complicate characterization making it time and cost-intensive. Integration of monitoring techniques during manufacturing will enable a thorough characterization of the component and will allow the detection of critical defects early on, ensuring the functionality and reliability necessary for industrial applications. This not only requires the adaption of non-destructive testing methods, but also of computational tools.

### 4.3. Scalable Characterization Methods: Resilience Is Key

Understanding the resilience of mechanical metamaterials will be key, should they become more than materials on the laboratory scale. Therefore, along with the design and development of mechanical metamaterials, suitable non-destructive characterization methods need to be developed. A combination of different classical characterization methods, together with advanced computational methods, will be needed to correlate the quality and functionality of parts. These multimodal methods will most likely not be identical for each metamaterial, as the properties of interest are different. A suitable toolbox of methods will set the foundation for scaling of mechanical metamaterials in industrial applications.

With complex materials like metamaterials, the conception of characterization strategies along the product life cycle will be essential.

Characterization during the manufacturing process is a very promising approach especially for additive manufacturing where the parts are built progressively. Monitoring techniques could capture flaws caused by manufacturing and other defects during production of the part, ideally providing an immediate three-dimensional representation of defects and material properties.

Characterization after manufacturing is equally important to characterize the finished good and as basis for monitoring techniques, as well as quality assurance that can be used in the applications to assess the remaining lifetime of materials.

Computational methods and algorithms will play an important role in the understanding of collected data and correlation to physical effects. Advanced methods of data fusion and the use of methods from artificial intelligence seem key to link data together in order to create meaningful information.

## 5. What Is Missing for the Deployment of Mechanical Metamaterials to Technical Applications

Mechanical metamaterials offer tremendous potential regarding the design space but require the establishment of new ways of developing and describing novel materials due to the structural hierarchies involved. The already complicated processing-microstructure-property relationship seems to become ever more challenging. However, one could also argue that properties are herewith decoupled from processing and the microstructure of the underlying material. The mechanical properties in mechanical metamaterials can be designed in a more controlled fashion through the mesoscopic structures of unit cells.

**Figure 6:**
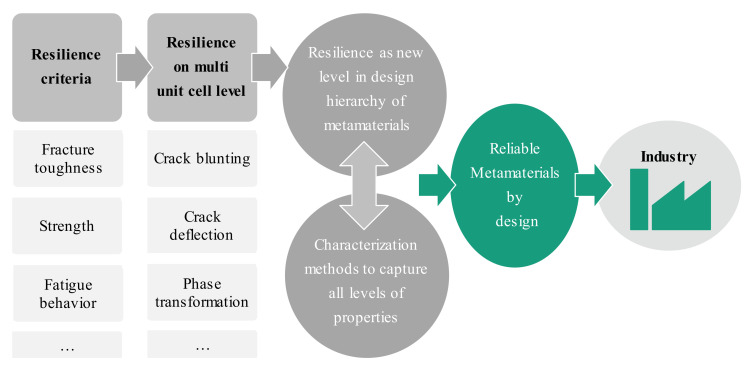
Key aspects to enable metamaterials for industrial applications: design for reliability.

Currently, the main objective of mechanical metamaterials research and development focuses on attaining the desired functionalities. While this approach shows the potential of mechanical metamaterials, many other aspects important for their usage in technical applications are still neglected. The reliability of mechanical metamaterials is a prerequisite for industrial applications and has not been the focus of research. To achieve reliable metamaterials, relevant properties such as strength, fracture toughness and fatigue behavior need to be considered (Figure 6). Most metamaterial designs in literature exhibit rather low fracture toughness when loaded in tension, as the unit cells cannot distribute the deformation in front of cracks into a quasi-infinite volume in form of plastic deformation, as described in classical fracture mechanics by Griffith or Paris [99,100,101]. Here, methods from classical microstructure design in ceramics need to be implemented in the design process in the future, e.g., crack deflection, crack blunting by micro crack formation, phase transformation or an embedded second phase with a higher toughness [102]. Resilience in mechanical metamaterials requires the implementation of another design hierarchy, translating the well-known effects from microstructure design to the mesoscopic multi unit cell level to achieve different modes of failure prevention through hierarchically structured materials.

Considering these properties is indispensable to produce metamaterials that are reliable and ready for industrial applications. All these characteristics are not only properties of a metamaterial system, a superposition of all its design properties as well as occurring defects. To enable metamaterials to revolutionize the industry, the design space needs to shift from the focus on pure functionality to include aspects of reliability and minimizing the effect of occurring defects.

The dream of revolutionizing industrial applications has not yet materialized. Considerably more effort is necessary to fully understand the interactions of different design parameters and defects within mechanical metamaterials and become an integral part in the product development process. Only then will new innovations based on metamaterials become reality in novel machines, transportation, lifestyle or household products.

## Figures and Tables

**Table 1:** Overview of different characterization methods for mechanical properties.

Type of Characterization	Material Type	Effects Studied	References
Compression tests	Shape memory polymers	Effects of different programming strains	[3]
	Acrylic Photoresin	Poisson’s ration, relaxation, recovery for varying holding times	[2]
		Poisson’s ratio for differently structured honeycomb-based structures	[16]
	Polymer	Change in mechanical properties upon changing the size of the unit cell	[42]
	Carbon nanotube reinforced PA12	Impact of structural alterations and varying density	[50]
	Silicone rubber	Impact of layer wise changing beam thickness	[41]
	Polymer	Deformation of rotation-based systems and influence of number of unit cells	[39]
Tensile tests	Polymer	Deformation of rotation-based systems and influence of number of unit cells	[39]
Three point bending tests	Shape memory polymers	Effects of different programming strains	[3]
DIC or similar	Different polymers	Visual tracking of deformations	[2,3,16,39,42]
